# Haptic Cues for Balance: Use of a Cane Provides Immediate Body Stabilization

**DOI:** 10.3389/fnins.2017.00705

**Published:** 2017-12-14

**Authors:** Stefania Sozzi, Oscar Crisafulli, Marco Schieppati

**Affiliations:** ^1^Centro Studi Attività Motorie, Istituti Clinici Scientifici Maugeri SPA SB, Institute of Pavia, IRCCS, Pavia, Italy; ^2^Department of Neuroscience, Rehabilitation, Ophthalmology, Genetics and Maternal Child Health, University of Genoa, Genoa, Italy; ^3^Department of Exercise and Sport Science, LUNEX International University of Health, Exercise and Sports, Differdange, Luxembourg

**Keywords:** sensorimotor integration, haptic sense, cane, standing balance, center of pressure, time to stabilization

## Abstract

Haptic cues are important for balance. Knowledge of the temporal features of their effect may be crucial for the design of neural prostheses. Touching a stable surface with a fingertip reduces body sway in standing subjects eyes closed (EC), and removal of haptic cue reinstates a large sway pattern. Changes in sway occur rapidly on changing haptic conditions. Here, we describe the effects and time-course of stabilization produced by a haptic cue derived from a walking cane. We intended to confirm that cane use reduces body sway, to evaluate the effect of vision on stabilization by a cane, and to estimate the delay of the changes in body sway after addition and withdrawal of haptic input. Seventeen healthy young subjects stood in tandem position on a force platform, with eyes closed or open (EO). They gently lowered the cane onto and lifted it from a second force platform. Sixty trials per direction of haptic shift (Touch → NoTouch, T-NT; NoTouch → Touch, NT-T) and visual condition (EC-EO) were acquired. Traces of Center of foot Pressure (CoP) and the force exerted by cane were filtered, rectified, and averaged. The position in space of a reflective marker positioned on the cane tip was also acquired by an optoelectronic device. Cross-correlation (CC) analysis was performed between traces of cane tip and CoP displacement. Latencies of changes in CoP oscillation in the frontal plane EC following the T-NT and NT-T haptic shift were statistically estimated. The CoP oscillations were larger in EC than EO under both T and NT (*p* < 0.001) and larger during NT than T conditions (*p* < 0.001). Haptic-induced effect under EC (Romberg quotient NT/T ~ 1.2) was less effective than that of vision under NT condition (EC/EO ~ 1.5) (*p* < 0.001). With EO cane had little effect. Cane displacement lagged CoP displacement under both EC and EO. Latencies to changes in CoP oscillations were longer after addition (NT-T, about 1.6 s) than withdrawal (T-NT, about 0.9 s) of haptic input (*p* < 0.001). These latencies were similar to those occurring on fingertip touch, as previously shown. Overall, data speak in favor of substantial equivalence of the haptic information derived from both “direct” fingertip contact and “indirect” contact with the floor mediated by the cane. Cane, finger and visual inputs would be similarly integrated in the same neural centers for balance control. Haptic input from a walking aid and its processing time should be considered when designing prostheses for locomotion.

## Introduction

Powered exoskeletons enable persons with various walking problems to ambulate over the ground. Several of these devices require the use of crutches to ambulate and maintain balance (Wang et al., 2015; see Asselin et al., 2016). Beyond their obvious mechanical effects (Bateni and Maki, 2005), crutches are a critical source of somatosensory inflow that provides information about body orientation with respect to the supporting surface through “extended physiological proprioception” (Simpson, 1974).

In this exploratory study, we asked whether the stabilizing effect on static balance of haptic information from a cane can be likened to that of haptic input from a light fingertip touch or to that of vision. It is well-known that haptic input reduces body sway during stance. Haptic input modifies spinal reflex excitability and the postural set even if it does not mechanically stabilize posture (Schieppati and Nardone, 1995; Jeka et al., 1996; Bove et al., 2006; Huang et al., 2009). The force exerted by the subjects onto a lightly touched stable frame need not be larger than 1 Newton (N) in order to induce the stabilizing effect (Kouzaki and Masani, 2008). The effect is similar to that obtained by opening the eyes with respect to standing eyes closed (Paulus et al., 1984; Sozzi et al., 2012; Honeine et al., 2015). Hence, addition of vision and haptic sense to the inherent proprioceptive inflow make the control of stance more effective (Jeka and Lackner, 1994, 1995; Sozzi et al., 2011, 2012; Honeine et al., 2015).

Haptic supplementation in elderly subjects or in patients with moderate to severe balance and gait impairment, as well as in blind subjects, is often dependent on their use of a cane (Jeka et al., 1996; Maeda et al., 1998; Hirahara et al., 2006; Albertsen et al., 2012; Guillebastre et al., 2012; Perreira et al., 2017; see Berglund, 2017, for an interesting point of view on the use of a cane). The cane would help these persons to compensate for their diminished cutaneous sensation from the feet (Peters et al., 2016) and to move independently while reducing their risk of falling, but it is normally used when standing as well, particularly in an unfamiliar environment or in presence of joint pain, or so. In people with neurological disorders of various nature, the cane is used to increase postural stability and to reduce the load on the weight–bearing lower extremities (Laufer, 2003; see Hamzat and Kobiri, 2008, for a complementary opinion).

The relevance of the supplementary haptic input for balance is further highlighted by the rapidity of the changes in sway as a consequence of adding or withdrawing the haptic or the visual information. Recently we estimated, in a population of young healthy subjects standing in tandem Romberg posture, the time-period necessary for the central nervous system to integrate the new sensory information and reweight its impact (or, in the case of withdrawal, to withstand the removal of the supplementary information and return to the proprioception-driven control; Sozzi et al., 2012; Honeine et al., 2015; see Honeine and Schieppati, 2014). In those experiments, the haptic information was a gentle touch (active or passive) of a firm surface exerted by the tip of the index finger or its removal by suddenly detaching surface and/or finger. The time necessary for the integration of haptic information, as assessed by the onset of the slightest detectable reduction in body sway, was >1 s, while that observed in the case of haptic withdrawal was significantly shorter. Next, a reweighting process led to a new dynamic steady-state in some 4–5 s in both cases. These time-intervals were not different from those measured by adding or withdrawing visual information (Sozzi et al., 2012).

With a cane, the haptic input is indirect, through a tool instead of by direct touch onto a stable frame with their own index finger. Sensory information would be produced by the cane touching the solid surface at some distance from the body, and by the subject being free to slide the cane on the ground. Conversely, in our previous investigations with finger touch, the solid surface touched by the fingertip was very close to the anterior surface of the trunk so that the vertical projection to the ground of this spot was at the border of the body's support surface defined by the feet position, and the wavering of the finger was very circumscribed (Sozzi et al., 2011, 2012; Honeine et al., 2015). The sensory input would also differ compared to that occurring on touching a frame with the finger because the perception of the contact would possibly rely on different sensory receptors. The contribution to the haptic input from upper limb muscle receptors (Rabin et al., 2008) would be perhaps more important for signaling the contact of the cane with the ground than the light-touch information from the skin of the fingertip.

Similar amounts of sway stabilization and similar latencies to stabilization for finger and cane touch would be in keeping with the hypothesis that an integration process is initiated by the haptic stimulus at the hand-cane interface, as if the fingers themselves touched a solid surface at the time-instant the cane touches the ground. A larger body sway and a longer latency to stabilization would speak instead of the need to include the computation of the actual location of the forearm and cane-tip touching-point into the reference frame for the control of body orientation in space. If the computation of the location of the touching point is necessary in order to reconstruct the image of the body-cane ensemble and calibrate the force of contact, stabilization might take more time with respect to when subject touches a solid surface with the fingertip directly.

We, therefore, assessed, in a population of normal young subjects standing in tandem feet position, the effect of cane use on body sway, and the time to stabilization or destabilization of balance, on adding or withdrawing the haptic input produced by the contact of the cane onto the ground. Our interest was three-fold: (a) to assess whether the use of the cane was indeed able to produce reduction of body sway, even if the cane was not fixed to the ground, and to measure the size of the effect; (b) to evaluate any effect of vision on the cane induced stabilization; (c) to estimate the latency at which the CNS incorporates the haptic information (or its withdrawal) connected with the cane stroke to the ground.

## Methods

### Subjects

Seventeen (7 males and 10 females) healthy subjects participated in this study. Their mean values (±standard deviation, SD) for age, weight and height were 25.7 years ± 6.6, 61.4 kg ± 10.6 and 167 cm ± 8. All procedures were carried out in accordance with the Declaration of Helsinki and with the adequate understanding and written informed consent of each subject. The ethics committee of the “Istituti Clinici Scientifici Maugeri” had approved the experiment (# 757 CEC).

### Task and procedures

Subjects stood in tandem position on a force platform (Kistler 9286BA), with the great toe of the rear foot immediately behind the heel of the front foot, with eyes closed or with eyes open. With EO, subjects were simply asked to look in front of them, and not to stare at any specific target. The visual scene of the laboratory walls at 6 m distance contained both horizontal and vertical profiles and sharp contours. Subjects chose which foot was the rear foot (it was the right foot in 12 subjects). The tandem posture was utilized to enhance medio-lateral sway (Sozzi et al., 2011, 2012, 2013; Honeine et al., 2015). Subjects were asked to hold with their dominant hand (right hand for all subjects) a straight plastic cane of 1 m length and 100 g weight, instrumented with a reflective marker fixed on the tip of the cane.

**Figure 1:**
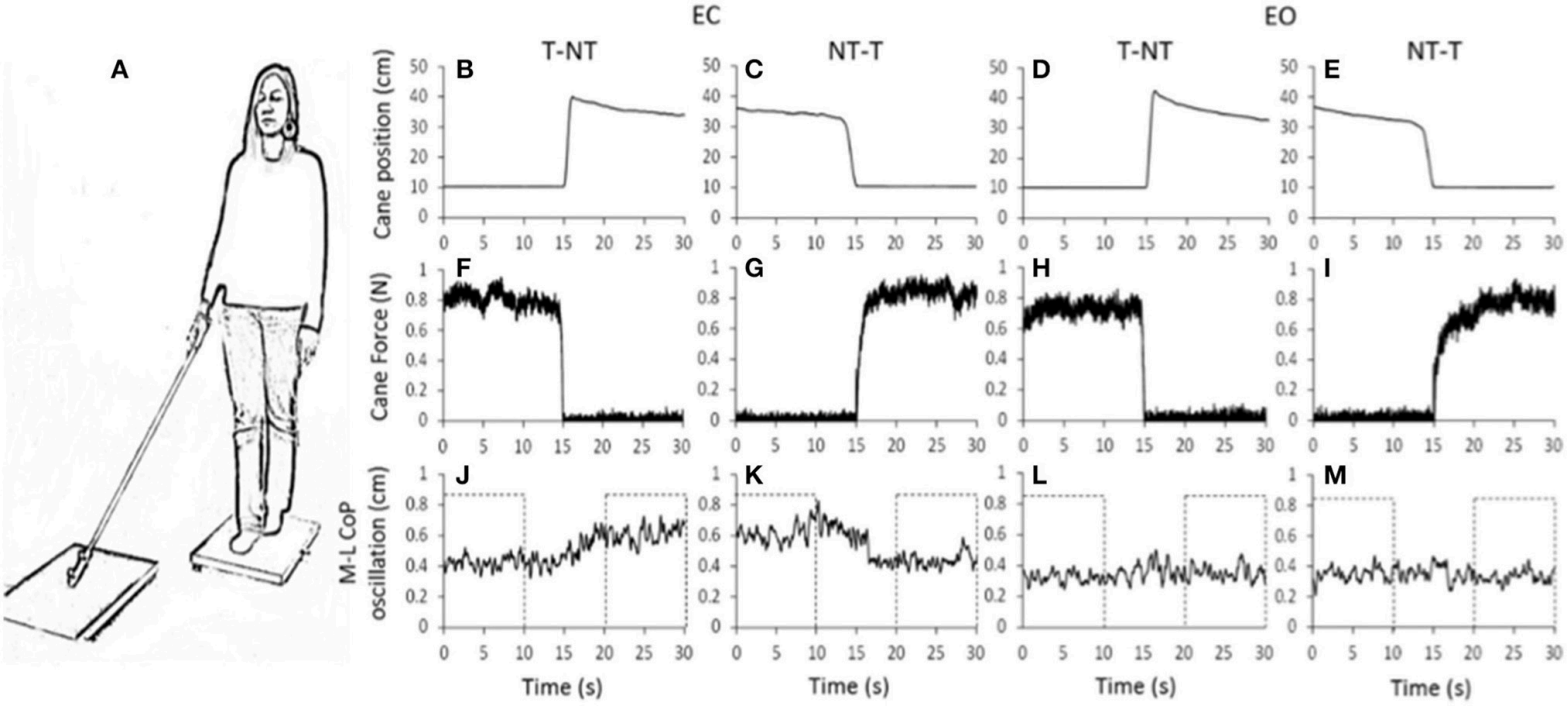
**(A)** Subjects stood with feet in tandem position on a force platform, with EC or EO, the cane resting on a second platform. **(B–M)** shows the mean value of the recorded signals in one representative subject. **(B–E)** vertical position of the marker placed close to the cane tip. **(F–I)** force applied by the cane onto the ground. **(J–M)** medio-lateral CoP oscillations, larger during EC than EO. With EC, after a short delay from the instant at which the cane was lifted, the oscillation amplitude increased (**J**, *t* = 15 s). Conversely, after the NT-T shift **(K)** the oscillation diminished. With EO **(L,M)**, no differences were obvious in the oscillations after the changes in haptic condition. The dotted boxes in **(J–M)** show the time intervals in which the oscillations were considered stationary and not affected by the shift in haptic condition.

After a verbal “go” signal given by the operator, subjects gently lowered the cane onto (or lifted it from) a second force platform equal to that mentioned above, which recorded the force applied by the cane. This force platform was placed in front of the subject and laterally spaced from the platform on which the subject stood (there was a distance of 55 cm from the center of the first to that of the second platform; Figure 1A). Successive “go” signals were given in a series, spaced by time intervals ranging each from 20 to 25 s, so that subjects periodically lowered the cane and withdrew it from the platform in sequence. A few practice trials were run to obtain touch forces on the platform smaller than 1 N. Subjects were asked not to move the cane in a reaction-time mode on hearing the verbal signal but to self-pace the movement necessary for lowering or lifting the cane from the ground when they felt so. Subjects underwent a series of at least 60 trials per direction of shift (Touch → NoTouch, T-NT; or vice versa NoTouch → Touch, NT-T) and per visual condition (EC or EO). Data were collected during 20 subsequent acquisition epochs of 240 s each (10 acquisition periods with EC and 10 with EO). Therefore, each epoch contained six haptic changes in which the cane was lowered onto the ground (NT-T) and six changes in which the cane was lifted (T-NT) from the ground. These epochs were then divided into trials, each of 30 s duration containing and centered on the change in haptic condition at *t* = 15 s. Then, equal-condition trials were aligned with the instant of the haptic shift and averaged. These big trial numbers were necessary in order to allow averaging of as many traces as possible in order to get consistent mean values for body oscillation and to reliably estimate the time following the shift in the sensory information, at which modifications occurred in body sway. Between each block of acquisition epochs, subjects were free to sit or move around for variable periods. The overall duration of the experimental session varied from 2 to 3 h, all conditions included.

### Center of foot pressure (CoP) and cane movement recording

Force signals from the two platforms were acquired at 140 Hz (SMART-D system, BTS, Italy). The output of the platform onto which the subject stood was the instantaneous position of the Center of foot Pressure (CoP) along the sagittal (antero-posterior, A-P) and the frontal plane (medio-lateral, M-L) during the standing trials. To quantify the amplitude of the CoP oscillations on the frontal and sagittal plane, the traces were high-pass filtered with a 2nd order Butterworth filter (cut-off frequency 0.1 Hz), and then rectified with a software developed in Labview (National Instruments, USA).

The mean level of force exerted by the cane on the ground was computed from the vertical force recorded by the platform onto which the cane rested. The position in space of the marker fixed on the tip of the cane was also acquired at 140 Hz by means of an optoelectronic device composed of 12 cameras (Smart-D, BTS, Italy) and stored in a PC for off-line analysis.

For each subject and trial, a cross-correlation (CC) analysis was performed between the traces of the cane tip and of the CoP M-L displacement. The CC coefficient (R) at time lag = 0 s was calculated by means of a software developed in Labview. A positive coefficient indicated an in-phase displacement of cane and CoP in the M-L direction, a negative coefficient indicated anti-phase displacement. The time lag was the time interval at which the absolute value of R was maximum. A negative time lag indicates that cane movement lagged the CoP movement.

### Mean level of CoP oscillation

For every trial recorded in each subject, the mean A-P and M-L oscillations of the CoP were computed under all haptic (NT and T) and visual (EC and EO) conditions at steady state. To this aim, each variable was averaged during the first and last 10 s periods of each trial containing a shift in sensory condition (see the dotted box in Figure 1). These periods did not contain the 10-s time interval centered on the sensory shift and were considered to be stationary and unaffected by the sensory shift (Sozzi et al., 2011, 2012; Honeine et al., 2015).

### Mean latency of change in body sway following the sensory shifts

The latencies of the changes in body sway following the haptic shift were measured only for the CoP oscillation in the frontal plane under EC condition. With EO, the effects of a shift in haptic information were small both in the frontal and sagittal plane. Further, even with EC, the presence or absence of haptic information influenced to a much larger extent the oscillation in the frontal than in the antero-posterior direction.

For each subject and condition of haptic shift (addition or withdrawal), we measured the latency following the sensory shift (cane put on the ground, NT-T) or cane off the ground (T-NT), at which M-L CoP oscillation diminished, or increased depending on the haptic-shift direction. Latency was estimated on the averaged traces of all the trials (*n* = 60) containing the sensory shift. Each successive mean value of the trace after the shift was compared to the mean value of the variable computed during the 15 s before the shift (reference value) by the one-sample Student's *t*-test with *n* = number of repetitions. The time after the shift, at which the *t*-value of the above comparisons bypassed the critical value corresponding to a 0.05 probability and remained above it for at least 100 ms, was taken as the time, at which the presence or absence of the haptic information began to affect the postural control mode (Sozzi et al., 2012; Schieppati et al., 2014).

### Statistical analyses

A 3-way repeated-measure ANOVA with direction of oscillation (M-L and A-P), presence or absence of haptic information (NT or T) and visual condition (EC and EO) was used to compare the mean levels of CoP oscillation calculated at steady state. The *post-hoc* analysis was made with Fisher's LSD test. The mean time-lags between cane and M-L CoP displacements were compared between visual conditions by paired Student's *t*-test. The mean latencies of the changes in M-L CoP oscillations with EC were compared between the two haptic-shift conditions by a paired Student's *t*-test. The software package used was Statistica (StatSoft, USA).

## Results

### Effect of the addition or withdrawal of haptic information on body sway

Figure 1 shows the averaged traces of the recorded signals of one representative subject standing on the force platform (Figure 1A) during the T-NT and NT-T trials under EC (Figures 1B,C,F,G,J,K) and EO condition (Figures 1D,E,H,I,L,M). The first-row panels show the vertical position of the marker placed on the tip of the cane. When the cane was on the ground, the force recorded by the platform (middle row panels) was <1 N under both visual conditions. The difference between visual conditions in the force applied by the cane was not significant (paired *t*-test, *p* = 0.10). The bottom-row panels show that, under the EC condition, following the T-NT shift (at time *t* = 15 s in all panels), the values of the oscillations (Figure 1J) increased after a short delay from the instant of the sensory shift. Conversely, when the cane was lowered onto the ground (NT-T), the values of the M-L CoP oscillations (Figure 1K) diminished in amplitude. Under the EO condition, there were negligible differences in M-L CoP oscillations (Figures 1L,M) following the shift in haptic condition, for either NT-T or T-NT direction of shift. All subjects, particularly in the EC condition, referred that when the cane rested on the ground they felt more stable than during the period in which there was no cane reference.

### Body sway under steady-state condition

**Figure 2:**
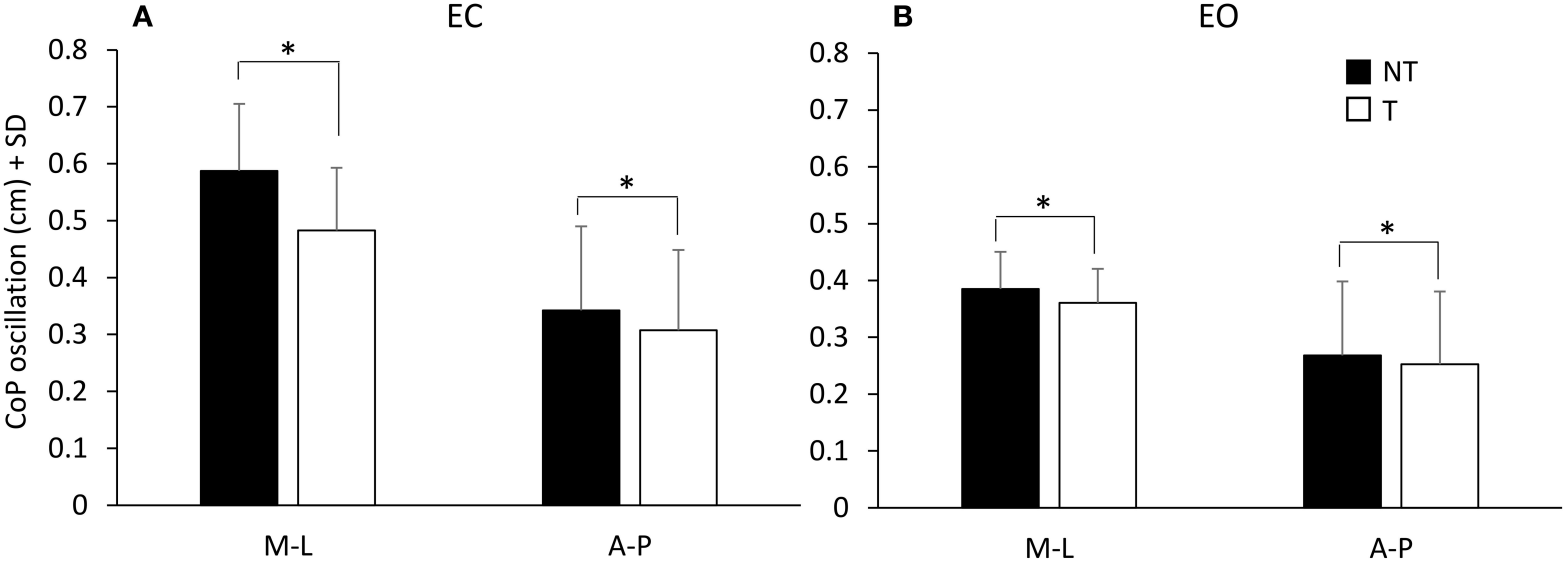
Mean values of CoP oscillations at steady-state under EC **(A)** and EO **(B)** condition. Black bars refer to no-touch (NT) condition, white bars to touch (T) condition. The CoP oscillations were greater along the frontal (M-L) than the sagittal (A-P) plane and greater under EC than EO condition. CoP oscillations were greater during the NT condition than during T condition. ^*^Indicates a significant difference (*p* < 0.05).

Figure 2 shows the mean values across subjects of the M-L and A-P oscillations of the CoP calculated at steady state under EC (Figure 2A) and EO (Figure 2B) conditions, with (T) or without (NT) the use of the cane. The CoP oscillations were greater along the M-L than the A-P direction [*F*_(1, 16)_ = 44.64, *p* < 0.001] during both the NT and the T condition (*post-hoc, p* < 0.05 for all comparisons).

There was a difference between the two visual conditions [EC vs. EO; *F*_(1, 16)_ = 76.73, *p* < 0.001] since the oscillations were larger with EC than EO (NT and T collapsed). Oscillations were also larger during the NT period (black bars) than during the T period (white bars) [NT vs. T; *F*_(1, 16)_ = 46.06, *p* < 0.001]. There was an interaction between direction of oscillation (M-L and A-P) and visual condition [*F*_(1, 16)_ = 13.23, *p* < 0.05], an interaction between direction of oscillation and presence or absence of haptic information [*F*_(1, 16)_ = 31.72, *p* < 0.001], and an interaction between direction of oscillation, visual condition and presence or absence of haptic information [*F*_(1, 16)_ = 18.13, *p* < 0.01]. In fact, the presence of haptic information diminished the CoP oscillation more under EC than EO condition and more in M-L than A-P direction. The Romberg quotients NT/T (EC) and EC/EO (NT) for the M-L CoP oscillations were 1.22 ± 0.13 and 1.54 ± 0.33 SD, respectively, indicating that both haptic input and vision reduced body sway. The haptic effect was, however, smaller than that of vision (paired *t*-test on the Romberg quotients, *p* < 0.001). With EO, the Romberg quotient NT/T (1.07 ± 0.09 *SD*) indicated no major haptic-induced stabilization compared to the haptic effect with EC (paired *t*-test on the Romberg quotients, *p* < 0.001).

### Cane-tip displacement follows body oscillation

**Figure 3:**
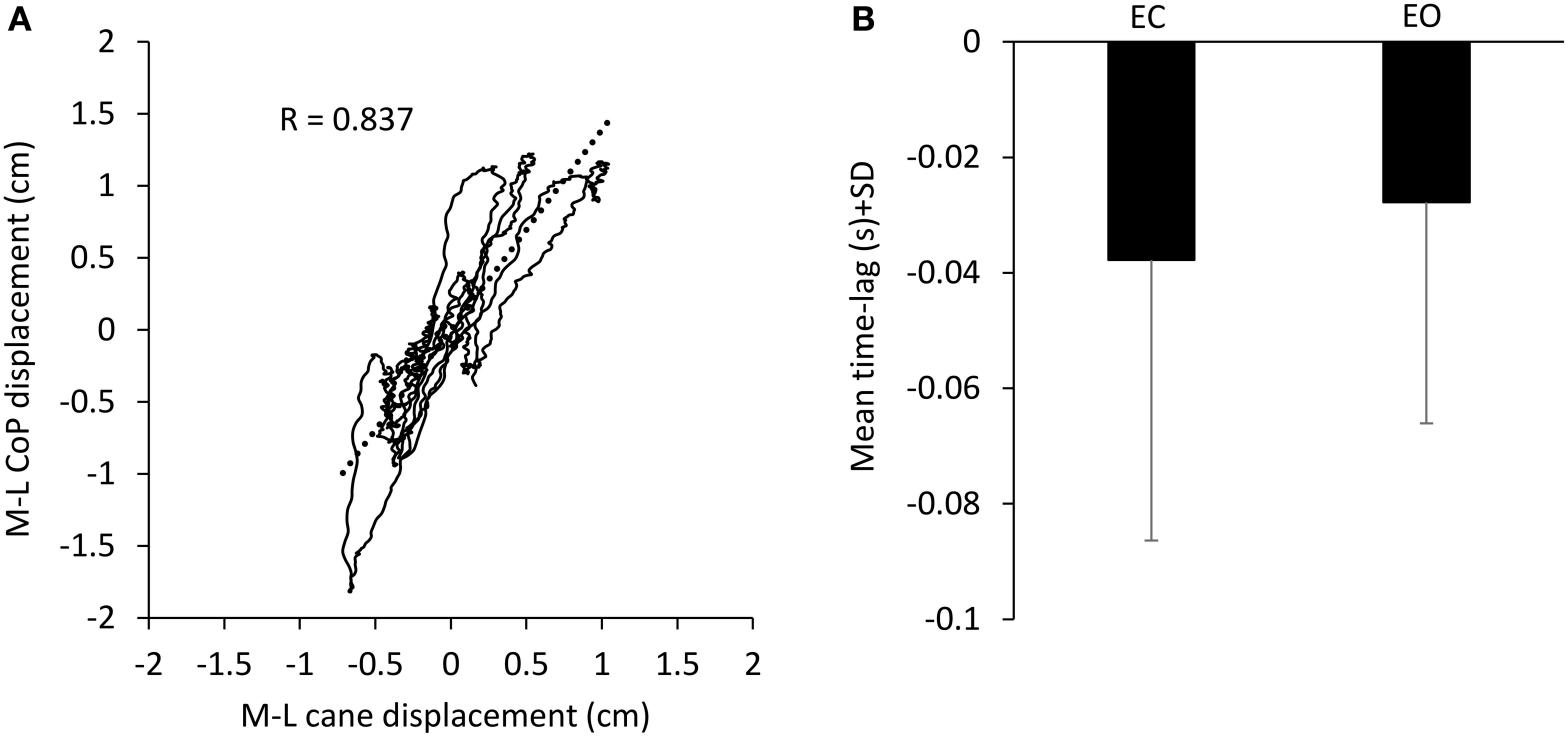
Association between CoP and cane displacement. **(A)** The displacement of the CoP along the frontal plane (M-L) was plotted against the cane movement in the same plane recorded during one 10-s period in which the cane rested on the ground in one subject EC. When the CoP moved to the right (positive values in the ordinate) also the cane moved to the right (positive values in the abscissa). **(B)** Mean time-lag between CoP and cane movement across subjects. Negative values of time-lag indicate that the cane movements lagged the CoP movements.

During the period in which the cane tip was resting on the ground, there was a good association between the movement of the cane and the CoP M-L oscillation (in the example reported in Figure 3A, *R* = 0.837, *p* < 0.001). When the CoP position moved to the right (positive values on the abscissa), also the cane moved to the right (positive values on the ordinate) and vice versa. The cane tip displacements were often smaller than those of the CoP. With EC, the CC coefficient calculated for the two traces was positive in the majority of subjects (15/17), ranging from −0.46 to 0.64 (all trials and subjects collapsed, mean *R* = 0.43 ± 0.34 SD). With EO, the CC coefficient was positive in the majority of the subjects as well, ranging from −0.38 to 0.68 (mean *R* = 0.47 ± 0.32 SD). Mean CC coefficients were slightly different between visual conditions (paired *t*-test, *p* < 0.05). The positive values of the CC coefficients indicate an overall in-phase movement of cane and CoP in the M-L direction. The mean time lag between cane and CoP displacement in the M-L direction (Figure 3B) was −37.7 ms ± 48.6 with EC and −27.7 ms ± 38.3 with EO (paired *t*-test, *p* = 0.3), indicating that the cane displacement lagged the CoP displacement regardless of the visual conditions.

### The delay from the sensory shift to the change in body sway is longer with addition than removal of haptic input

The latencies of the changes in body sway on touching the ground with the cane or lifting it off ground were estimated for each subject on the mean M-L CoP oscillation trace under EC condition. The reason was that the changes in the levels of CoP oscillations, caused by the addition or removal of haptic information, were much greater in the M-L than A-P direction.

**Figure 4:**
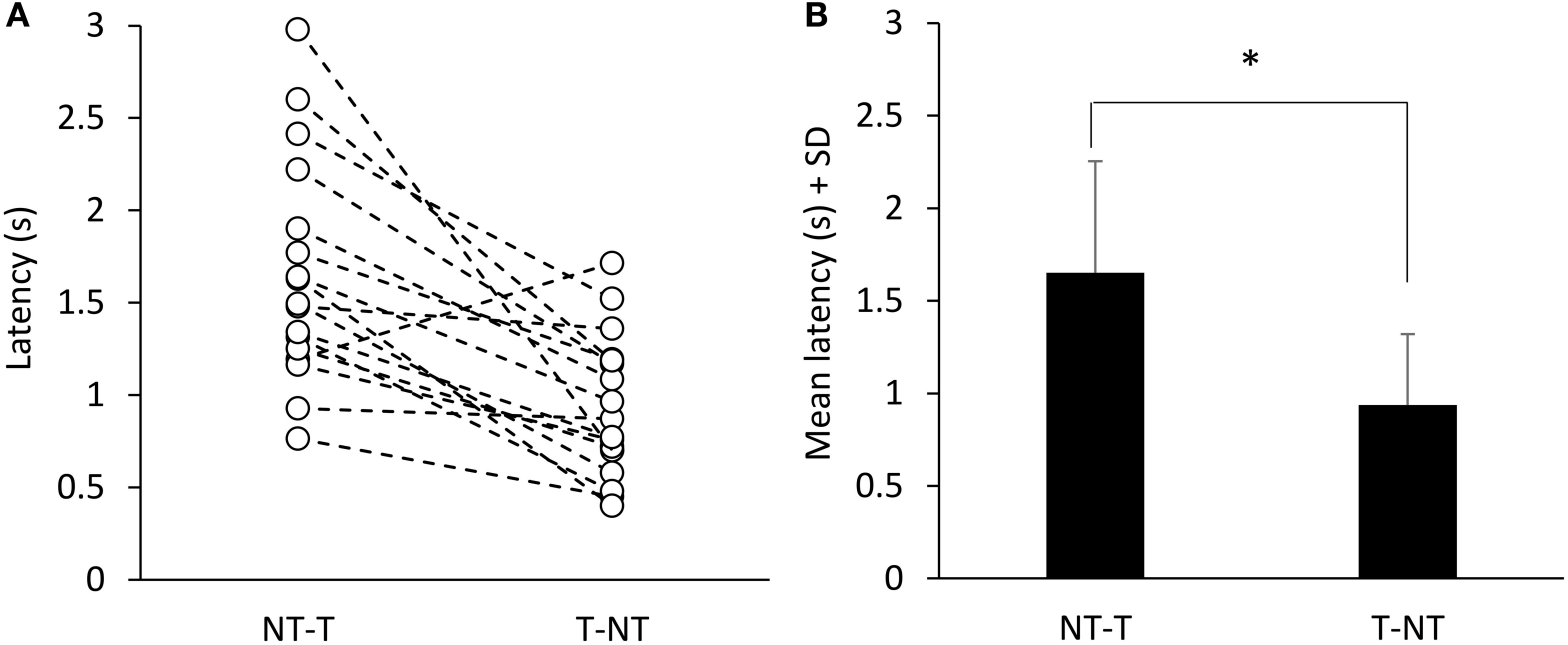
Time intervals from haptic change to change in body oscillation when standing with EC. **(A)** Latencies of the changes in M-L CoP oscillations calculated for each subject. **(B)** Mean latencies across subjects. The latencies were longer for the NT-T shift than for the T-NT shift. ^*^Indicates a significant difference (*p* < 0.001).

Figure 1 showed that, with a short delay following the shift in haptic condition (at *t* = 15 s), the oscillations increased when the cane was removed (T-NT condition) or decreased when the cane was put on the ground (NT-T condition). The latencies of the changes in oscillations calculated for each subject are reported in Figure 4A for the NT-T and for the T-NT condition. Latencies ranged from 0.76 to 2.98 s for the NT-T shift in haptic condition and from 0.45 to 1.7 for the T-NT shift, and were longer for the addition than for the withdrawal of haptic information. In Figure 4B, the mean latencies across subjects for the two sensory shifts are reported. Mean latencies were longer by about 0.7 s for the NT-T (1.64 s ± 0.6 SD) than for the T-NT condition (0.93 s ± 0.4 SD) (paired *t*-test, *p* < 0.001).

## Discussion

We investigated the changes occurring in the body sway of young healthy subjects standing in tandem position and performing the task of lowering a cane onto the ground or lifting it. We were specifically interested in estimating the latency at which body sway diminishes in response to the haptic input connected with the cane contact onto the ground (or at which sway increases on removing the cane).

In line with previous reports (Sozzi et al., 2011, 2012; Honeine et al., 2015), the CoP oscillations were smaller with EO than EC. With EC, oscillations were smaller with cane touch (T) compared to no touch (NT). With EO cane had little effect. All subjects felt more stable when the haptic input was available, and referred that standing in tandem was easier with than without the haptic input. The latencies to the initial changes in oscillation in response to the sensory shift, assessed on the EC data, were longer after addition (NT-T, about 1.6 s) than withdrawal (T-NT, about 0.9 s) of the haptic input.

It is well-known that standing humans sway less when vision is allowed (Straube et al., 1994; Sozzi et al., 2011; Sarabon et al., 2013). Haptic information from light finger touch produces body sway reduction as well (Jeka and Lackner, 1994; Jeka et al., 1996; Lackner et al., 1999; Bolton et al., 2011; Sozzi et al., 2012; Kanekar et al., 2013; Schieppati et al., 2014; Honeine et al., 2015). Hence, minor displacement of the visual field on the retina, or touch information from the fingertip, not granting mechanical stabilization (Lackner et al., 2001; Kouzaki and Masani, 2008), are sufficient for consistently reducing body sway. These effects are obvious when balance is critical, like standing with the feet in tandem position (Huang et al., 2009; Sozzi et al., 2011, 2012; Sarabon et al., 2013; Honeine et al., 2015). The visual and haptic information would be little exploited under quiet stance with feet parallel, but become relevant when balancing under unfavorable conditions (De Nunzio and Schieppati, 2007; Sozzi et al., 2011). Visual or haptic input add to the proprioceptive information, to the cutaneous input from the foot soles, and to the vestibular input (Mergner et al., 2009; Billot et al., 2013; Bronstein, 2016). Thus, in spite of the visual and haptic inputs being just a fraction of the overall sensory inflow, they are not at all negligible for stabilization (Lackner et al., 1999).

### Similar stabilization by a cane and fingertip touch

Here, we used a cane as a way for decreasing body sway during eyes-closed stance. This tool proved to be effective for stabilization, even when the cane exerted <1 N force on the ground, and even when the cane was not fixed to the ground, as in other investigations on this issue (Jeka et al., 1996; Maeda et al., 1998; Albertsen et al., 2010; Ustinova and Langenderfer, 2013; Oshita and Yano, 2016). Of note, the experiments were conducted under tandem stance, which is a challenging sway situation (Sozzi et al., 2013), in order to clearly detect differences between the condition with/without cane touch. Quiet stance with feet parallel would have required a much larger number of repetition to detect the onset of sway changes on adding/removing the haptic input, owing to the sensitivity of the analytical method.

In our study, the cane was free to move, as it would happen under natural circumstances, and its displacement on the force platform accompanied the oscillations of the center of foot pressure. Interestingly, it appeared that the cane was not used as a pivot to control body sway, exerting a force onto the ground in order to move the body; contrary, its translation normally followed the displacement of the center of foot pressure by some 40 ms (see Figure 3). This further suggests that the cane moves with the swaying body and that stabilization can be obtained through the haptic reference rather than using the cane as a mechanical support (Misiaszek et al., 2016). Whether such effect can transfer to different conditions, such as postural disturbances (Owings et al., 2000), or to reactive balance control (Schinkel-Ivy et al., 2016), or to walking (Rabin et al., 2015) is an important issue, worth specific investigation.

### The effect of vision and the interaction with haptic input

The reduction in the center of foot pressure oscillation with the cane resting on the ground under EC condition (diminution to 82%) was smaller than that obtained with vision (when standing without a cane, the diminution in sway amplitude on opening the eyes reached 67%). In a similar vein, the effect obtained with the cane was also somewhat smaller than that obtained by using the fingertip, as shown in a previous investigation (Sozzi et al., 2012; fingertip touch reduced sway to about 75%, no touch compared to touch, eyes closed). This value had been obtained in a different population of subjects matched for age and gender, but having comparable sway values eyes-closed under no-touch condition. Interestingly, roughly similar stabilizing effects of the haptic input have been also noted as a consequence of touching either a rough or a slippery surface (Jeka and Lackner, 1995).

When the cane was used while vision was allowed (eyes open all the time), the concurrent haptic information produced little extra reduction of the body sway compared to no-cane, as already observed (Albertsen et al., 2012), as if vision would largely overrule the haptic information provided by the cane (Krishnan and Aruin, 2011). The use of the cane did decrease body sway in the M-L plane with eyes open, but this decrease was limited as if an occlusion effect ensued. This would occur if haptic inflow and vision would share common mechanisms for stabilization. Besides, the steadying effect of vision was large, and the use of the cane (not exerting any mechanical action) may not easily further reduce the oscillations when subjects are standing in tandem. Not alternatively, oscillations would not diminish beyond a certain value, because a given body-sway stimulus is in any case required for activating proprioceptors and labyrinth and sending meaningful information to the brain (van Emmerick and van Wengen, 2000; Carpenter et al., 2010). But then again, in this case, a given body sway would also return a certain valuable input from the moving cane tip. The effect of haptic input from the cane would be a substitute for vision and become crucial during stance or walking in blind subjects or in sighted subjects with eyes closed, when absence of vision produces clear-cut effects on the spatial characteristics of gait requiring an increased computational brain effort (Oates et al., 2017; Oliveira et al., 2017). Of note, vision and touch reduce muscle activity and co-contraction of antagonist leg muscles, which is characteristic of the tandem stance (Sozzi et al., 2012, 2013), much as vision does during another challenging balance condition, such as counteracting continuous postural perturbations (Sozzi et al., 2016).

### Sensori-motor integration time

The congruence of haptic input from cane and visual input in enhancing body stability under steady-state condition had a counterpart in the substantial equivalence of the latency for the changes in body oscillation, on addition or on withdrawal of the corresponding sensory inputs. In the previous investigations mentioned above, though, the mean latency to stabilization onset on opening the eyes was about 1.2 s, hence just shorter than that following cane touch to the ground (they were instead equal on removing vision or touch; Sozzi et al., 2011, 2012; Honeine et al., 2015). However, the mean latencies to decrease and increase of sway (about 1.4 and 1 s, respectively), observed when the haptic input originated from light fingertip touch (Sozzi et al., 2012; Honeine et al., 2015) were similar to those measured in the present study. Overall, these data speak in favor of a substantial equivalence of the haptic information derived from a “direct” contact of fingertip with a stable surface and of the “indirect” contact with a stable surface mediated by the cane, and confirm a slightly longer latency for the integration of haptic compared to visual information.

### The effect of the haptic information from the cane is not affected by the interaction with the task execution

The profile of the changes in body sway induced by the haptic information (or by its withdrawal) seemed not to be affected by the movement necessary to lift or lower the cane onto the ground. This finding and conclusion were not anticipated, because movements of upper limb and hand for lowering or lifting the cane, though limited in extent and velocity, might have interfered with the exploitation of the haptic sensory input (Saradjian, 2015). Further, these movements might have produced anticipatory or corrective postural adjustments, possibly impeding appropriate reweighting of the new haptic sensory inputs because of other types of balance priorities (such as counteracting the task of gently lifting or lowering the cane). Or, the onset of the changes in oscillation might have been simply concealed by large CoP displacements connected to those tasks. The experiment was not designed for sorting out potentially interfering effects of anticipatory or corrective postural adjustments: however, these were rarely obvious in the individual CoP traces and disappeared on averaging multiple epochs. This points to small and asynchronous postural adjustments, and to the rather constant latency of the sway reducing (or increasing) effects of the changes in haptic input. Hence, we would suggest that the reweighting phenomenon must be robust, and possibly represents a priority for the postural control system, independent of the possible modulation of the haptic input itself, connected with the voluntary act of moving the cane, as occurs for other inputs along the thalamo-somatosensory cortex pathway (Bolton et al., 2012; Seki and Fetz, 2012; Song and Francis, 2015; Colino et al., 2017). In this light, these findings lend support to the conclusions of Saradjian et al. (2013) and Mouchnino et al. (2015) that the brain exerts dynamic control over the transmission of the afferent signals according to their current relevance during a critical balance condition.

### Processing time and neural circuits: open questions

Regardless of the modality and origin of the sensory information (at least for visual and haptic—finger or cane), the nervous system seems to react within roughly similar time intervals. It might be conjectured that the entry to the “posture stabilizing” centers is common to both visual and haptic inputs, and independent of any parallel pathway conveying inputs for different physiological functions. Admittedly, time intervals pertaining to different modalities may not be stated definitely in our test paradigm. The paradigm and analytical procedures are certainly accurate for detecting differences between addition and withdrawal of a sensory input, but may not detect subtle differences connected with the peripheral traveling and central processing of information from different sources.

The relatively long integration time may be an expression of the computation for shifting to a less energetically expensive pattern of stance control. The latency to change in the oscillation pattern is much longer than a reflex (~50 ms), or a startle reaction (~100 ms), or else a quick voluntary response (~150 ms), and is even longer than the balance-correcting responses triggered by a perturbation of stance (~200 ms) (Valls-Solé et al., 1999; Grüneberg et al., 2005; Sozzi et al., 2012; Honeine and Schieppati, 2014). Interestingly, the latencies to stabilization onset were somewhat longer but of the same order of magnitude as the time lag between motor command and body sway, estimated by means of cross-correlation analysis of leg muscle EMG activities and body sway size in subjects standing quietly without support (Masani et al., 2011). These authors found that the longer the time lag of the cross-correlation (up to half a second) the smaller the body sway, and concluded that a control strategy producing a longer preceding time for the motor command can stabilize the body more effectively. It is not unlikely that the values we found for latency to stabilization might have implied activation of the processes mentioned in Masani et al. (2011).

Processing of the haptic input for balance stabilization would be subserved by dedicated cortical networks, possibly at parietal cortex level (Kaulmann et al., 2017), while the more rapid shift toward a new state of increased sway on withdrawing the haptic input would be produced at subcortical level. Anticipated loss of balance (lifting the cane) would allow for the cortical pre-selection and optimization of brain stem postural activity (Jacobs and Horak, 2007; see Shadmehr, 2017). Removal of sensory inputs equally rapidly triggers a “default” reaction of the posture stabilizing centers to the sudden withdrawal of the critical haptic originating from finger and cane contact or vision, whereby body sway quickly shifts to a larger oscillation pattern (Sozzi et al., 2012; Honeine and Schieppati, 2014; Assländer and Peterka, 2016; Honeine et al., 2017). This is the consequence of the lack of critical information on the one hand and a condition for stronger proprioceptive and vestibular stimulation on the other.

### Balance, locomotion, and neural prostheses for locomotion

We would, therefore, argue that haptic input as provided by using a cane is sufficient for improving balance, almost as a gentle fingertip touch of a stable structure or vision of the surrounding space. Hence, cane or crutches would provide an information which can be typically processed by the nervous system along with other, more “natural” (tactile, visual, vestibular) inputs. It is arguable that haptic input from such devices can be exploited not only during stance, but also during locomotion, all the more so when locomotion is aided by neural prostheses. Haptic inflow from cane would be crucial during gait initiation and cooperate with the anticipatory postural adjustments in helping weight distribution between both legs so as to produce the best stability conditions for optimal gait initiation (Caderby et al., 2017). In this line, Chastan et al. (2010) have mentioned the relevance of the somatosensory input for balance control during gait initiation. Further insight on the mechanisms of action of any haptic input finalized to reducing the oscillation of the center of pressure during the successive stance phases of walking would be welcome.

Walking velocity is clearly affected by postural instability in several clinical conditions, as in cerebellar and neuropathic diseases (Morton and Bastian, 2003; Nardone et al., 2009, 2014) or in patients with stroke (Hsiao et al., 2017), chronic obstructive pulmonary disease (Morlino et al., 2017) or Parkinson's disease (Giardini et al., submitted). Investigation on the timing of haptic-motor integration should be extended to a larger population of normal young subjects, to elderly persons and to visually impaired and neurological populations, such as Parkinson's disease patients (Rabin et al., 2015). The findings of these investigations would prove useful in the design of new rehabilitation devices. Haptic control is needed for any locomotion exoskeleton, where step production consists of discrete shifts from one posture to another. Implementation of an appropriate time-lag between changes in haptic inflow and their effects on balance control would represent an important aspect of the design of the control system for exoskeletons (see Mergner, 2007; Peterka, 2009; O'Doherty et al., 2012). In a broader context, it is not unlikely that somatosensory prosthetics may help not only perception and action (O'Doherty et al., 2011; Tyler, 2015), but also contribute to creating an appropriate response in the domain of the control of the equilibrium. The findings of the present investigation might foster implementation of new technologies taking into account the “natural” time constraints of multi-modality sensory integration, and represent a step toward the building of biologically inspired balance- and locomotion devices.

## Author contributions

SS: contributed with data collection, data analysis, and drafted the manuscript; OC: contributed with data collection and data analysis; MS: contributed with project creation, data analysis, and wrote and edited the manuscript.

### Conflict of interest statement

The authors declare that the research was conducted in the absence of any commercial or financial relationships that could be construed as a potential conflict of interest.
